# Uncovering Potential Druggable Targets in Coronary Atherosclerosis: A Proteome‐Wide Mendelian Randomization Study With Cross‐Platform and Experimental Validation

**DOI:** 10.1155/cdr/7359278

**Published:** 2026-08-29

**Authors:** Da Gao, Haiyan Lin, Shengjie Wang, Yanwei Wang, Hao Yang, Zicheng Wang

**Affiliations:** ^1^ Department of Cardiovascular Medicine, Ningbo Medical Center Lihuili Hospital, Ningbo, Zhejiang, China, nbws.gov.cn

**Keywords:** colocalization analysis, coronary atherosclerosis, Mendelian randomization, plasma proteins, SMR analysis

## Abstract

**Background:**

Coronary atherosclerosis (CA) is a leading cause of cardiovascular morbidity and mortality worldwide. This study is aimed at identifying candidate plasma proteins and potential therapeutic targets for CA.

**Methods:**

We performed a proteome‐wide Mendelian randomization (MR) analysis using integrated protein quantitative trait loci (pQTLs) and genome‐wide association study (GWAS) summary data. Forward two‐sample MR was first performed using cis‐pQTLs from the UK Biobank Pharma Proteomics Project (UKB‐PPP), followed by reverse MR analysis to exclude potential reverse causality. Bayesian colocalization analysis was conducted to ensure that the associations between proteins and CA were driven by shared genetic variants. Summary‐data‐based MR (SMR) combined with HEIDI testing was used to prioritize proteins and eliminate linkage bias. Significant proteins were cross‐referenced with a curated druggable genome to identify their potential therapeutic relevance. Cross‐platform validation was performed using the SomaScan‐based pQTL dataset from deCODE genetics. An oxidized low‐density lipoprotein (ox‐LDL)–induced human umbilical vein endothelial cell (HUVEC) injury model was used for the evaluation of prioritized proteins.

**Results:**

Forward MR analysis using UKB‐PPP cis‐pQTL data identified 45 CA‐associated proteins (23 protective, 22 risk‐related; FDR < 0.05), with no reverse causality observed. Five proteins—PARP1, SDCCAG8, FST, FOLH1, and NCAN—were prioritized through MR (FDR < 0.05), colocalization analysis (PP.H4 > 0.50), and SMR analysis with HEIDI filtering (*p*_SMR < 0.05; *p*_HEIDI > 0.05). All five proteins were included in the druggable genome list, supporting their potential therapeutic relevance. PARP1 showed consistent associations across Olink and SomaScan platforms and was upregulated in an ox‐LDL‐induced HUVEC model as assessed by western blot.

**Conclusions:**

This study identified PARP1, SDCCAG8, FST, FOLH1, and NCAN as genetically prioritized candidate proteins for CA, with PARP1 showing the most consistent evidence across analyses. Further validation and mechanistic studies are warranted.

## 1. Introduction

Coronary atherosclerosis (CA) is the underlying pathological process of coronary artery disease, the leading cause of mortality and morbidity worldwide [1]. According to the Global Burden of Disease study, atherosclerotic cardiovascular diseases account for nearly one‐third of all global deaths [2, 3]. Despite advancements in pharmacological treatments and revascularization techniques, a considerable residual risk remains, underscoring the urgent need for novel preventive and therapeutic strategies [4].

The development of CA involves a complex interplay of lipid accumulation, endothelial dysfunction, inflammatory responses, and immune system dysregulation [1, 5]. Whereas therapies that lower low‐density lipoprotein cholesterol (LDL‐C), such as statins and proprotein convertase subtilisin/kexin Type 9 (PCSK9) inhibitors, have significantly reduced cardiovascular events, the high cost and requirement for long‐term injections of PCSK9 inhibitors may decrease patient compliance [6, 7]. Anti‐inflammatory therapies, such as interleukin‐1*β* (IL‐1*β*) inhibitors exemplified by the CANTOS trial, have also shown clinical benefits but are associated with increased risk of infections [8]. Moreover, plaque instability and atherosclerotic progression are driven not only by lipid accumulation but also by persistent inflammatory and immune mechanisms, which are not sufficiently targeted by existing therapies [9]. Therefore, exploring novel molecular pathways and identifying new druggable targets are critical steps toward improving the management of CA.

Circulating plasma proteins play central roles in lipid metabolism, immune regulation, endothelial function, and vascular remodeling [10]. Their accessibility in peripheral blood and involvement in key atherogenic processes make them attractive candidates for biomarker discovery and therapeutic targeting [10, 11]. For example, therapies directed at PCSK9 have successfully translated plasma protein biology into clinical benefit [12]. Additionally, biomarkers such as oncostatin M (OSM) and growth differentiation Factor 15 (GDF‐15) have been associated with plaque vulnerability and cardiovascular risk [13, 14]. However, many proteins identified by traditional observational studies have not been confirmed as causal mediators due to limitations including confounding factors and reverse causality.

Mendelian randomization (MR) offers a powerful approach that uses genetic variants as instrumental variables (IVs) to assess causal relationships between exposures and outcomes [15]. Integrating protein quantitative trait loci (pQTLs) data with genome‐wide association study (GWAS) enables proteome‐wide MR analyses, allowing systematic evaluation of thousands of plasma proteins for their causal associations with disease development [16]. In addition, Bayesian colocalization analysis and summary‐data‐based Mendelian randomization (SMR) with Heterogeneity in Dependent Instruments (HEIDI) testing help strengthen causal inference by distinguishing shared causal variants from confounding due to linkage disequilibrium (LD) or horizontal pleiotropy [17]. Recent studies have demonstrated the effectiveness of these approaches. Islam et al. [18] have identified 45 candidate biomarker genes potentially causally associated with stroke risk, providing valuable insights into the functional genes related to stroke through MR and colocalization analysis. In the cardiovascular field, Liu et al. [19] have applied a tissue‐specific expression quantitative trait locus (eQTL)–driven MR framework and identified vesicle‐associated membrane Protein 8 (VAMP8), milk fat globule‐EGF Factor 8 (MFGE8), and platelet‐derived growth Factor D (PDGFD) as potential therapeutic targets for CA. Furthermore, a study by Xie et al. [20] has found two mitochondrial‐related genes, UQCC1 and ETFDH, to be genetically associated with sarcopenia, highlighting the role of mitochondrial dysfunction in the disease pathogenesis.

Building upon these advances, we performed a comprehensive proteome‐wide MR analysis to explore plasma proteins potentially involved in CA pathogenesis, aiming to identify potential biomarkers and potential therapeutic targets for future investigation.

## 2. Methods

### 2.1. Study Design

**Figure 1:**
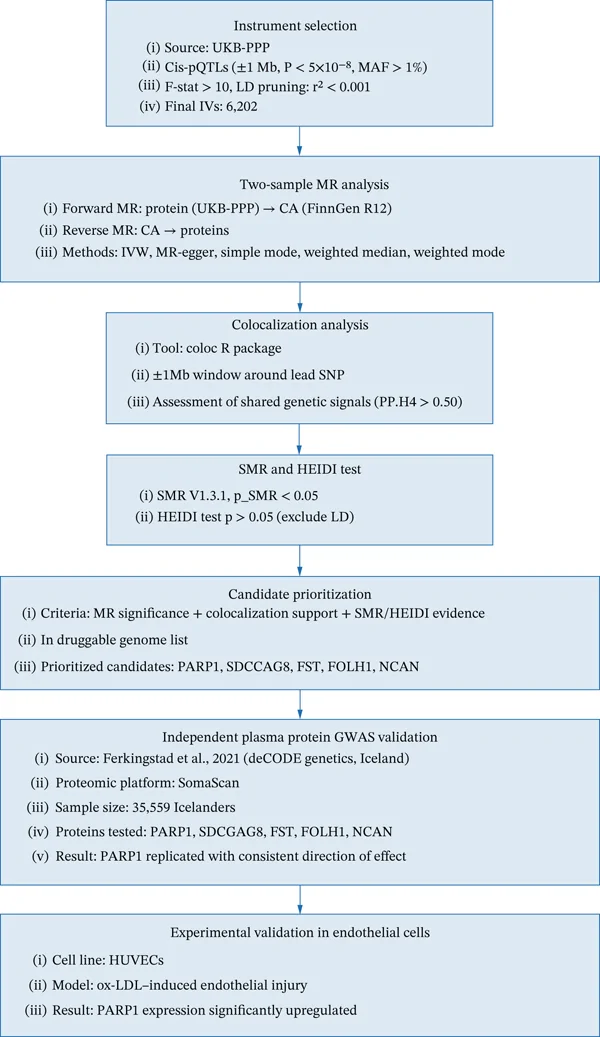
Flowchart of the study design.

This study was conducted to systematically identify potential druggable plasma proteins associated with CA. To achieve this objective, we applied a comprehensive analysis framework integrating proteome‐wide MR, reverse MR, Bayesian colocalization analysis, and SMR with HEIDI testing. GWAS summary statistics for CA were obtained from FinnGen R12, whereas plasma pQTL datasets were derived from the UK Biobank Pharma Proteomics Project (UKB‐PPP). In addition, cross‐platform validation and preliminary in vitro functional experiments were performed to further support the robustness and biological relevance of prioritized candidate proteins. An overview of the complete analytical workflow is illustrated in Figure 1, which depicted the sequential steps undertaken in the study. All datasets were derived from open‐access sources and contained deidentified summary‐level data. Therefore, no additional ethical approval was required for this analysis.

### 2.2. Selection of IVs

pQTL summary statistics were derived from the UKB‐PPP, which profiled over 2900 plasma proteins using the Olink platform across approximately 54,306 individuals of European ancestry [21]. Cis‐pQTLs were defined as genetic variants located within ±1 Mb of the transcription start or end site of the corresponding gene [22]. Only variants achieving genome‐wide significance (*p* < 5 × 10^−8^) and exhibiting a minor allele frequency greater than 1% were retained to ensure robust association signals and sufficient statistical power. Variants located within the extended major histocompatibility complex region (Chromosome 6: 25–34 Mb) were excluded due to complex LD patterns. Further LD pruning was conducted using the European reference panel from the 1000 Genomes Project, retaining only independent variants with a pairwise *r* ^2^ < 0.001 within a 10 Mb window. Allele harmonization was performed to ensure consistent alignment of effect alleles across exposure and outcome datasets, and palindromic SNPs with ambiguous strand orientation were excluded. Following these filtering steps, a total of 6202 genome‐wide significant and LD‐independent cis‐pQTL variants were selected as IVs for two‐sample MR analysis.

All IVs used in MR were required to satisfy three core assumptions [23]: (i) a strong association with the exposure (plasma protein levels), (ii) independence from any confounding factors that could influence both the exposure and the outcome, and (iii) an exclusive effect on CA risk through their impact on the exposure pathway, without alternative causal routes. Instrument strength was evaluated using the F‐statistic, and variants with F‐statistics ≤ 10 were excluded to minimize weak instrument bias [24]. In addition, the IV selection strategy was specifically designed to reduce the likelihood of violating assumptions (ii) and (iii) [23]. In particular, restricting instruments to cis‐acting pQTLs, excluding variants within the extended major histocompatibility complex region, and applying stringent LD pruning were intended to minimize confounding and horizontal pleiotropy, thereby reducing the probability that selected IVs act as trans‐pQTLs for other CA‐relevant proteins. To further address assumptions (ii) and (iii), all candidate cis‐pQTLs were systematically screened against publicly available databases, including PhenoScanner and the GWAS Catalog, to exclude variants showing genome‐wide significant associations with known CA risk factors or unrelated traits [25].

### 2.3. Proteome‐Wide MR Analysis

To evaluate the potential causal effects between circulating plasma protein levels and the risk of CA, we performed two‐sample MR analyses in both forward and reverse directions. The exposure data for forward MR consisted of cis‐pQTL summary statistics derived from the UKB‐PPP, whereas the outcome data were obtained from FinnGen R12 (https://r12.finngen.fi/pheno/I9_CORATHER), using the phenotype finngen_R12_I9_CORATHER, which includes 63,307 cases and 416,171 controls. Disease definitions were based on ICD‐10 Codes I24, I25, T82.2, and Z95.1 (https://risteys.finngen.fi/endpoints/I9_CORATHER). In reverse MR, GWAS‐significant SNPs associated with CA served as the exposure, and genetically predicted protein levels from UKB‐PPP were treated as the outcome. Genetic variants significantly associated with protein abundance (cis‐pQTLs) were selected as IVs for two‐sample MR analysis.

All MR analyses were conducted in R Version 4.2.2 using the TwoSampleMR (V0.6.6) package. SNPs were harmonized across exposure and outcome datasets to align alleles and eliminate ambiguous palindromic variants. For proteins with a single instrumental SNP, causal estimates were calculated using the Wald ratio method. For proteins with multiple independent SNPs, the primary causal estimates were obtained using the IVW method under a multiplicative random effects model [26]. To evaluate heterogeneity among instruments, we used Cochran′s *Q* test, and MR‐Egger regression was performed to detect and adjust for directional horizontal pleiotropy, with the MR‐Egger intercept interpreted only when significant. Additional sensitivity analyses included the weighted median, simple mode, and weighted mode estimators to confirm the robustness of the findings. The MR Steiger test was used to confirm that the genetic instruments explained more variance in the exposure than in the outcome, thereby validating the assumed causal direction. Leave‐one‐out and single‐SNP analyses were also performed to ensure that individual variants did not disproportionately influence the causal estimates. To control for multiple testing, false discovery rate (FDR) correction was applied, with a significance threshold set at *p* < 0.05.

### 2.4. Colocalization Analysis

To confirm whether the same genetic variant underlies both the protein expression and disease association signal, we performed Bayesian colocalization analysis using the coloc R package (V5.1.0). Bayesian colocalization analysis was performed to determine whether the same genetic variant is responsible for associations between plasma protein levels and CA risk [27]. For each protein–trait pair that showed evidence of association in the MR analysis, we extracted SNP‐level summary statistics within ±1000 kb of the lead cis‐pQTL variant. This analysis was conducted for five mutually exclusive hypotheses [28]: PP.H0, indicating no association with either trait; PP.H1, association with protein levels only; PP.H2, association with CA only; PP.H3, association with both traits but driven by distinct causal variants; and posterior probability for H4 (PP.H4), association with both traits driven by a shared causal variant. Strong evidence of colocalization was defined as a PP.H4 greater than 0.80, indicating a high likelihood that the same variant influences both circulating protein abundance and CA risk. PP.H4 values between 0.50 and 0.80 were interpreted as moderate evidence supporting a shared genetic signal, whereas PP.H4 values below 0.50 were interpreted as providing limited evidence of colocalization.

### 2.5. SMR Analysis

To further prioritize genetically implicated proteins and reduce confounding due to LD, we performed SMR using the SMR software (V1.3.1). This method tests whether the effect of a cis‐pQTL on CA risk is mediated through the same variant that regulates protein abundance [29]. For each protein–trait pair identified in MR, the top‐ranked cis‐SNP was used as the IV in SMR analysis. To further differentiate between pleiotropy and LD‐driven associations, we applied the HEIDI test across local SNPs in the region. HEIDI *p* > 0.05 indicates evidence consistent with a shared causal variant [30]. Only protein–disease pairs that passed both SMR and HEIDI criteria were considered genetically prioritized candidate proteins and retained for subsequent interpretation and druggability assessment.

### 2.6. Sensitivity Analyses

To evaluate the robustness of our findings, we conducted multiple sensitivity analyses. Leave‐one‐out analysis was used to test the influence of individual SNPs by iteratively excluding one instrument and recalculating the MR estimate. Single‐SNP analysis was performed to visually assess outliers. Proteins meeting the MR and SMR significance thresholds, passing HEIDI testing, and showing at least moderate colocalization support were retained as candidate proteins for further evaluation.

### 2.7. Selection of Druggable Plasma Proteins Based on pQTL Data

To systematically evaluate potential therapeutic targets for CA, we selected druggable plasma proteins and their corresponding genetic instruments based on large‐scale proteomic and genomic datasets [31]. A comprehensive reference list of druggable proteins was compiled based on previously published classification systems, including the framework proposed by Finan et al. [32], which has incorporated targets of FDA‐approved drugs as well as those currently under active clinical investigation. During the MR and SMR analyses, plasma proteins showing significant associations with CA were cross‐referenced with this druggable protein list. Proteins that met the criteria for statistical significance (FDR < 0.05), passed HEIDI testing (*p*_HEIDI > 0.05), and demonstrated at least moderate colocalization support (PP.H4 > 0.50) were considered genetically prioritized candidate proteins for further evaluation.

### 2.8. Cross‐Platform Validation Using SomaScan‐Based pQTL Dataset From deCODE Genetics

To further evaluate the robustness of the primary findings and to account for potential platform‐specific effects, we performed an independent cross‐platform validation of the associations identified in the primary Olink‐based pipeline by examining whether genetically predicted protein levels showed consistent directional genetic associations with CA. Summary statistics were obtained from the large‐scale Icelandic proteomics GWAS conducted by deCODE genetics and reported by Ferkingstad et al. [31], which quantified 4907 plasma proteins in 35,559 individuals of European ancestry. In this validation analysis, cis‐acting pQTLs were extracted from the SomaScan dataset using the same genomic definition applied in the primary analysis. Instrument selection criteria were aligned with those used in the UKB‐PPP‐based analysis. This cross‐platform comparison was conducted to assess the reproducibility of protein–CA associations across different proteomic platforms, without re‐estimating a full proteome‐wide MR framework.

### 2.9. Cell Culture and Treatment

Human umbilical vein endothelial cells (HUVECs) were obtained from the Cell Bank of the Chinese Academy of Sciences (Shanghai, China) and cultured in endothelial cell medium supplemented with 10% fetal bovine serum and 1% penicillin–streptomycin. Cells were maintained at 37°C in a humidified incubator with 5% CO_2_, and the culture medium was replaced every 2–3 days. For establishment of an oxidized low‐density lipoprotein (ox‐LDL)–induced endothelial injury model, HUVECs were seeded into 6‐well plates and allowed to reach approximately 70%–80% confluence. Cells were then treated with ox‐LDL at concentrations of 25, 50, or 100 *μ*g/mL for 24 h, whereas cells treated with vehicle alone (0 *μ*g/mL ox‐LDL) served as the control group, as previously described [33]. After treatment, cells were collected for subsequent analyses.

### 2.10. Enzyme‐Linked Immunosorbent Assay (ELISA)

The concentrations of IL‐1*β*, IL‐6, and tumor necrosis factor‐alpha (TNF‐*α*) in cell culture supernatants were quantified using commercially available ELISA kits (Jingmei Biotechnology, Jiangsu, China). Briefly, cell culture supernatants were collected and centrifuged at 3000 × g for 10 min at 4°C to remove cellular debris. The clarified supernatants were then subjected to ELISA analysis. After the final reaction step, absorbance was measured at 450 nm using a microplate reader (Bio‐Rad Inc., United States).

### 2.11. Cell Viability Assay

Cell viability was evaluated to identify the appropriate concentration range of ox‐LDL using a Cell Counting Kit‐8 (CCK‐8; Solarbio, Beijing, China). HUVECs were plated into 96‐well plates at a density of 5 × 10^3^ cells per well and subjected to the indicated treatments. Following treatment, 10 *μ*L of CCK‐8 reagent was added to each well and incubated at 37°C for 2 h. Absorbance was recorded at 450 nm using a microplate reader (BioTek, United States).

### 2.12. Western Blot

Total proteins were extracted using ice‐cold RIPA lysis buffer supplemented with a protease inhibitor cocktail. Lysates were incubated on ice for 30 min and then centrifuged at 12,000 × g for 15 min at 4°C. The supernatants were collected, and protein concentrations were determined using a BCA assay kit (Beyotime Biotechnology, Shanghai, China). Equal amounts of protein (30 *μ*g) were separated on 8%–12% SDS‐PAGE gels and transferred onto PVDF membranes using a semidry transfer system. After blocking with 5% nonfat milk for 1 h at room temperature, membranes were incubated overnight at 4°C with primary antibodies against poly(ADP‐ribose) Polymerase 1 (PARP1) (1:200, sc‐74470, Santa Cruz Biotechnology, United States), intercellular adhesion Molecule 1 (ICAM1) (1:1000, 10831‐1‐AP, Proteintech, United States), vascular cell adhesion Molecule 1 (VCAM1) (1:1000, 11444‐1‐AP, Proteintech, United States), and GAPDH (1:5000, 60004‐1‐Ig, Proteintech, United States). After washing with TBST, membranes were incubated with HRP‐conjugated secondary antibodies (goat antimouse IgG‐HRP, A0192; goat antirabbit IgG‐HRP, A0208; Beyotime Biotechnology, Shanghai, China) for 1 h at room temperature. Protein bands were visualized using an enhanced chemiluminescence detection kit (Thermo Fisher Scientific, United States).

### 2.13. Statistical Analysis

All data are expressed as the mean ± standard deviation (SD) from three independent experiments. Differences among multiple groups or between two groups were assessed using one‐way ANOVA followed by Tukey′s post hoc test or Student′s *t*‐test. A *p* value < 0.05 was considered statistically significant. Statistical analyses and graph generation were conducted using GraphPad Prism 9.0 (GraphPad Software Inc., San Diego, California).

## 3. Results

### 3.1. MR Identified 45 Plasma Proteins Associated With CA

**Figure 2:**
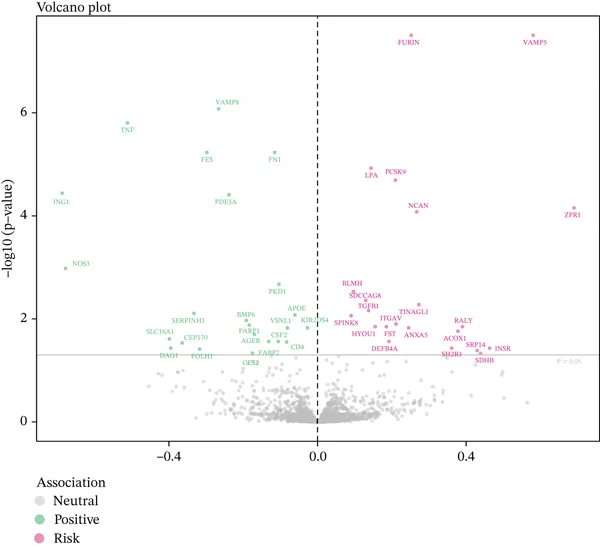
Volcano plot of proteome‐wide MR analysis for CA. The volcano plot displays the results of the proteome‐wide MR analysis. The *x*‐axis represents the beta coefficients (effect sizes) for each protein, and the *y*‐axis shows the −log_10_‐transformed *p* values. Proteins significantly associated with increased risk are highlighted in green, whereas those inversely associated with risk are highlighted in red. Nonsignificant associations are shown in gray. The horizontal gray line represents the nominal significance threshold (*p* = 0.05), and the vertical dashed line indicates a beta of zero. MR, Mendelian randomization.

**Figure 3:**
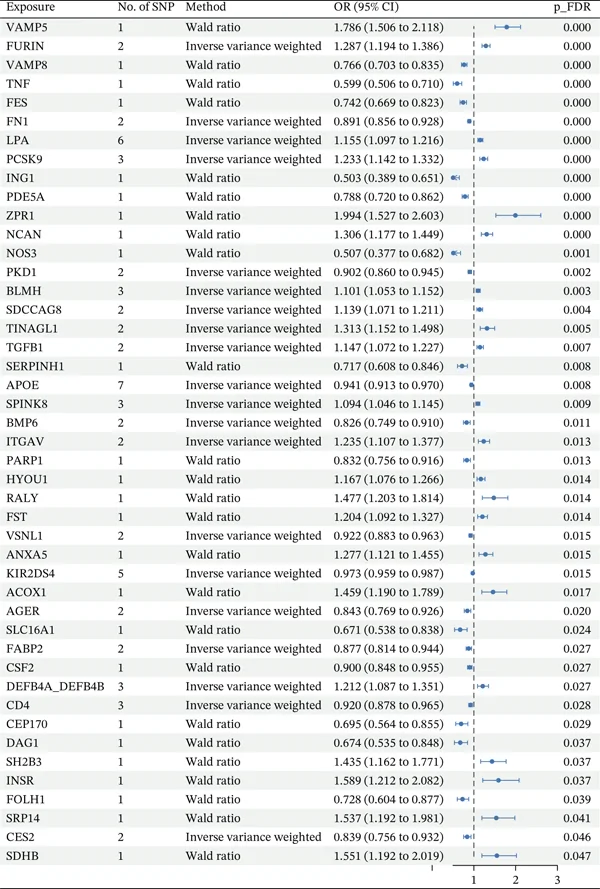
Forest plot of 45 plasma proteins associated with CA. The forest plot summarizes odds ratios (ORs) and 95% confidence intervals (CIs) from the MR analysis for all 45 proteins with *p* < 0.05. MR, Mendelian randomization.

To explore the associations between circulating protein levels and CA, we conducted a proteome‐wide MR analysis using cis‐pQTLs as IVs. A total of 6202 genome‐wide significant and LD‐independent cis‐pQTL variants were selected as IVs (Table S1). The results of the volcano plot (Figure 2) and the forest plot (Figure 3) showed that a total of 45 plasma proteins were significantly associated with CA after FDR correction (FDR < 0.05), including 23 protective‐ and 22 risk‐associated proteins. Among the 23 protective‐associated proteins, several showed strong inverse associations. These included VAMP8 (OR = 0.766; 95% CI: 0.703–0.835; *p* = 8.33 × 10^−7^), TNF (OR = 0.599; 95% CI: 0.506–0.710; *p* = 1.57 × 10^−6^), FES (OR = 0.742; 95% CI: 0.669–0.823; *p* = 5.87 × 10^−6^), FN1 (OR = 0.891; 95% CI: 0.856–0.928; *p* = 5.87 × 10^−6^), ING1 (OR = 0.503; 95% CI: 0.389–0.651; *p* = 3.63 × 10^−5^), and PDE5A (OR = 0.788; 95% CI: 0.720–0.862; *p* = 3.90 × 10^−5^). Additional protective proteins included NOS3, PKD1, SERPINH1, BMP6, PARP1, APOE, SLC16A1, CEP170, DAG1, folate Hydrolase 1 (FOLH1), FABP2, AGER, CSF2, CD4, VSNL1, KIR2DS4, and CES2, all with ORs < 1 and FDR‐adjusted *p* < 0.05.

In contrast, 22 proteins were positively associated with CA risk. Among them, VAMP5 (OR = 1.786; 95% CI: 1.506–2.118; *p* = 3.12 × 10^−8^) and FURIN (OR = 1.287; 95% CI: 1.194–1.386; *p* = 3.12 × 10^−8^) were the most significant. Other risk‐associated proteins included PCSK9, LPA, ZPR1, neurocan (NCAN), BLMH, serologically defined colon cancer Antigen 8 (SDCCAG8), TINAGL1, TGFB1, SPINK8, ITGAV, HYOU1, follistatin (FST), INSR, RALY, ANXA5, ACOX1, SH2B3, SRP14, DEFB4A_DEFB4B, and SDHB, all with ORs > 1 and *p* < 0.05. To evaluate the robustness of the MR findings, a series of sensitivity analyses were performed. Heterogeneity tests showed no substantial evidence of between‐instrument heterogeneity for the prioritized protein–CA associations (Table S2). MR‐Egger intercept tests did not indicate significant directional horizontal pleiotropy (Table S3). Leave‐one‐out analyses demonstrated that the overall MR estimates were not driven by any single SNP (Table S4). Single‐SNP analyses were conducted for associations with multiple IVs to evaluate the consistency of MR estimates across individual SNPs (Table S5).

### 3.2. Reverse MR Did Not Indicate an Effect of CA on the 45 Plasma Proteins

**Figure 4:**
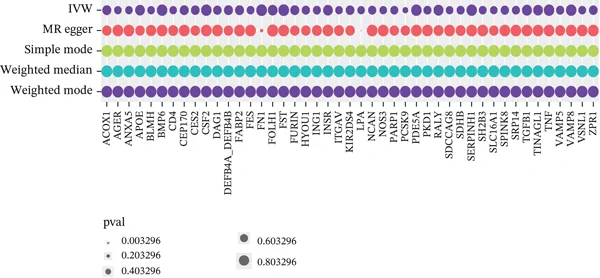
Bubble plot showing *p* values from reverse MR analyses of 45 plasma proteins. Each column represents a protein, and each row represents one MR method: IVW, inverse‐variance weighted; MR‐Egger, Egger regression; simple mode, unweighted modal estimator; weighted median, median‐based estimator; weighted mode, mode‐based estimator with weight adjustment. Larger, lighter‐colored bubbles represent higher *p* values.

To assess whether CA is associated with changes in plasma protein levels, we performed reverse MR analysis. In this analysis, CA was treated as the exposure, and the 45 proteins identified in the forward MR were used as outcomes. Five MR methods were applied, including IVW, MR‐Egger, weighted median, weighted mode, and simple mode. The bubble plot (Figure 4) showed that all proteins had *p* values greater than 0.05 in the IVW model. These findings suggest that CA is unlikely to influence the levels of these proteins.

### 3.3. Colocalization Analysis Supported Shared Genetic Signals for Multiple Proteins

**Figure 5:**
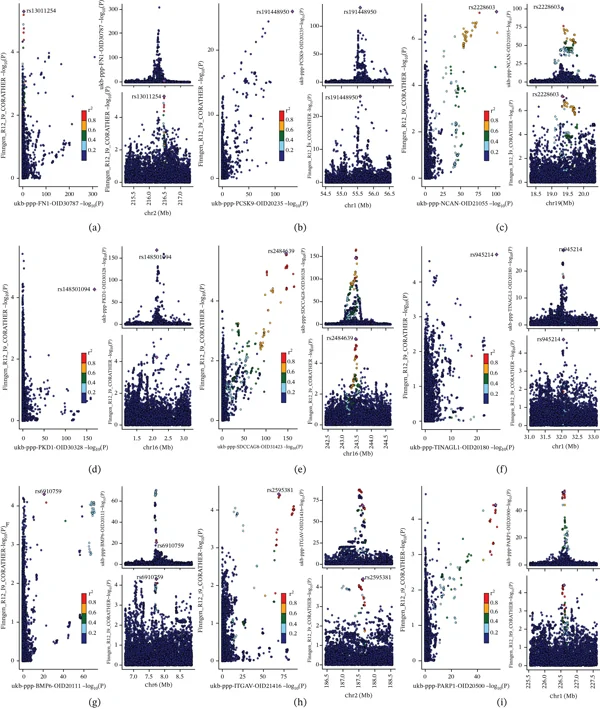
Colocalization plots for nine proteins with strong evidence from MR analyses. Each picture showed the regional association signals of the protein quantitative trait locus (pQTL, *x*‐axis) and CA GWAS (*y*‐axis) within ±500 kb of the lead SNP: (a) FN1, (b) PCSK9, (c) NCAN, (d) PKD1, (e) SDCCAG8, (f) TINAGL1, (g) BMP6, (h) ITGAV, and (i) PARP1. SNPs are colored according to linkage disequilibrium (*r* ^2^) with the lead variant. The colocalization posterior probability (PP.H4) indicates the likelihood that both traits share the same causal variant.

To further evaluate whether the associations identified by MR were supported by shared genetic signals, we performed Bayesian colocalization analysis across candidate protein loci. Among the proteins analyzed, 13 proteins demonstrated varying degrees of colocalization support. Of these, nine proteins—PCSK9, NCAN, PKD1, SDCCAG8, TINAGL1, ITGAV, PARP1, BMP6, and FN1—exhibited strong colocalization evidence with PP.H4 > 0.80 (Figure 5). Additionally, HYOU1 and FOLH1 demonstrated moderate support for colocalization (0.65 < PP.H4 < 0.80), whereas FST and INSR showed relatively lower but still moderate support (0.50 < PP.H4 < 0.65), suggesting less robust but still potentially relevant evidence for a shared genetic signal. Detailed posterior probability values (PP.H0–PP.H4) for all 13 proteins are presented in Table S6. Overall, these colocalization findings strengthen the evidence supporting these protein–CA associations and provide additional insight into their genetic regulatory architecture.

### 3.4. SMR and HEIDI Analyses Identified Five Genetically Prioritized Candidate Proteins

**Figure 6:**
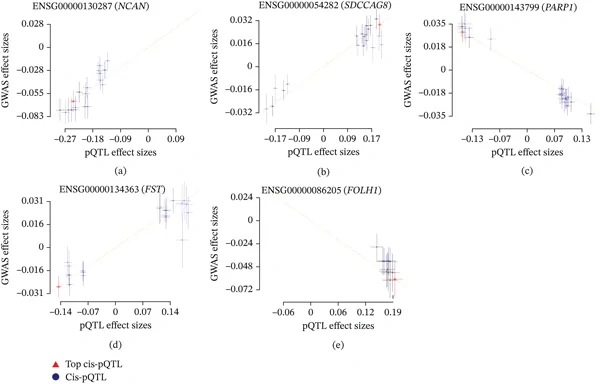
SMR and HEIDI analyses identified five genetically prioritized candidate proteins. Scatter plots from SMR and HEIDI analyses for five candidate plasma proteins prioritized through complementary genetic analyses: (a) NCAN, (b) SDCCAG8, (c) PARP1, (d) FST, and (e) FOLH1. Each point represents a cis‐pQTL SNP used as an instrumental variable, with the *x*‐axis showing the effect size on plasma protein levels (pQTL effect) and the *y*‐axis showing the effect size on CA risk (GWAS effect). Red points indicate the top associated cis‐pQTL for each protein, whereas blue points represent nearby cis‐pQTLs. The dashed orange line represents the fitted regression line under the SMR model.

To further evaluate the associations between plasma protein levels and CA, we performed SMR analysis combined with HEIDI testing. SMR analysis evaluated whether a genetic variant influencing protein abundance was also associated with disease risk through a shared genetic signal, whereas the HEIDI test was used to distinguish horizontal pleiotropy from confounding due to LD. A total of 14 proteins were subjected to SMR analysis. Detailed SMR and HEIDI results for all 14 proteins are provided in Table S7. Among them, five proteins satisfied the stringent criteria of *p*_SMR < 0.05 and *p*_HEIDI > 0.05. These proteins were NCAN (*p*_SMR = 1.02 × 10^−6^, *p*_HEIDI = 0.3106), SDCCAG8 *p*_SMR = 4.09 × 10^−5^, *p*_HEIDI = 0.3427, PARP1 $\begin{array}{c} \left(p\_\text{SMR}=2.480.5751\times 10^{-4},p\_\text{HEIDI}=\right) \end{array}$, FST $\begin{array}{c} \left(p\_\text{SMR}=2.630.8204\times 10^{-4},p\_\text{HEIDI}=\right) \end{array}$, and FOLH1 $\begin{array}{c} \left(p\_\text{SMR}=1.390.2934\times 10^{-3},p\_\text{HEIDI}=\right) \end{array}$. These five proteins consistently showed statistically significant associations between genetically predicted protein levels and CA risk, without evidence of heterogeneity, suggesting that their effects are unlikely to be explained by LD alone (Figure 6). Together with the MR and colocalization analyses, these findings supported their prioritization as candidate proteins for further evaluation, although the strength of colocalization evidence differed among proteins. In contrast, the remaining proteins, including TINAGL1, PCSK9, ITGAV, FN1, BMP6, HYOU1, PKD1, INSR, and TGFB1, either failed the HEIDI test (indicating possible linkage confounding) or did not reach statistical significance in SMR analysis, and were therefore not retained as prioritized candidate proteins.

### 3.5. SomaScan‐Based Validation Highlighted PARP1 as a Consistently Associated Protein

**Figure 7:**
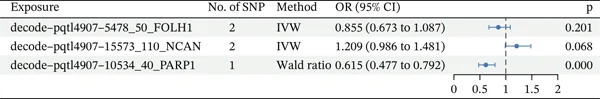
Cross‐platform validation of selected candidate plasma proteins using the SomaScan pQTL dataset. Forest plot showing Mendelian randomization estimates for the associations between genetically predicted plasma protein levels and CA based on SomaScan‐derived cis‐pQTL instruments from the Icelandic proteomics study. Odds ratios (ORs) and 95% confidence intervals (CIs) are shown.

In the SomaScan‐based pQTL dataset from deCODE genetics, the forest plot (Figure 7) demonstrated a statistically significant association between PARP1 and CA using a single cis‐pQTL instrument under the Wald ratio method, with an OR of 0.615 (95% CI: 0.477–0.792, *p* = 1.65 × 10^−4^), indicating a protective effect that was directionally consistent with the primary Olink‐based analysis. FOLH1 showed an OR of 0.855 (95% CI: 0.673–1.087, *p* = 0.201), whereas NCAN showed an OR of 1.209 (95% CI: 0.986–1.481, *p* = 0.068). Whereas the associations for FOLH1 and NCAN did not reach statistical significance in the independent SomaScan dataset, the observed effect directions were consistent with the primary analysis. In addition, cross‐platform validation was not feasible for SDCCAG8 due to the absence of corresponding SomaScan measurements and for FST due to an insufficient number of eligible cis‐pQTL variants to support MR analysis.

### 3.6. PARP1 Was Upregulated in ox‐LDL‐Treated HUVECs

**Figure 8:**
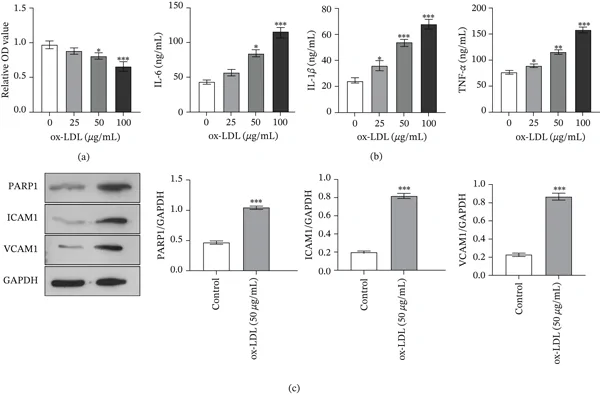
PARP1 was upregulated in ox‐LDL‐treated HUVECs (*n* = 3 per group). (a) Cell viability after different concentrations of ox‐LDL (25, 50, and 100 *μ*g/mL) or vehicle alone (0 *μ*g/mL ox‐LDL) was evaluated using the CCK‐8 assay. (b) Levels of IL‐1*β*, IL‐6, and TNF‐*α* in HUVECs treated with different concentrations of ox‐LDL were measured by ELISA. (c) Protein expression levels of VCAM1, ICAM1, and PARP1 in HUVECs treated with ox‐LDL (50 *μ*g/mL) or vehicle control were examined by western blotting. Data are presented as mean ± SD from three independent experiments.  ^∗^ *p* < 0.05;  ^∗∗^ *p* < 0.01;  ^∗∗∗^ *p* < 0.001.

Based on the above integrative analyses, PARP1 was selected for experimental validation in an ox‐LDL‐induced endothelial injury model of CA. To establish this in vitro model, HUVECs were exposed to increasing concentrations of ox‐LDL for 24 h. CCK‐8 assays showed that treatment with 25 *μ*g/mL ox‐LDL had no significant effect on cell viability, whereas exposure to 50 and 100 *μ*g/mL ox‐LDL resulted in a significant reduction in cell viability (Figure 8a). As shown in Figure 8b, ox‐LDL stimulation induced a concentration‐dependent increase in the secretion of the proinflammatory cytokines IL‐6, IL‐1*β*, and TNF‐*α*, with significantly elevated levels observed at 50 and 100 *μ*g/mL compared with the vehicle‐treated control group, and the highest levels detected at 100 *μ*g/mL. Although 100 *μ*g/mL ox‐LDL induced the highest levels of inflammatory cytokines, it was also associated with a more pronounced reduction in cell viability. Therefore, 50 *μ*g/mL ox‐LDL was selected for subsequent experiments to achieve robust endothelial activation while minimizing the loss of viable cells. Western blot analysis revealed that ox‐LDL treatment (50 *μ*g/mL) markedly increased the protein expression levels of the endothelial activation markers ICAM1 and VCAM1, confirming successful induction of endothelial activation (Figure 8c). Importantly, PARP1 protein expression was also significantly upregulated in ox‐LDL‐treated HUVECs compared with the control group (Figure 8c).

## 4. Discussion

In this study, using an integrative analytical framework that incorporated MR analysis based on UKB‐PPP pQTL data and CA GWAS summary statistics, Bayesian colocalization, SMR/HEIDI analyses, and druggable genome annotation, we identified five genetically prioritized candidate proteins for CA—PARP1, SDCCAG8, FST, FOLH1, and NCAN. Among these, PARP1 showed consistent associations across Olink and SomaScan proteomic platforms and was upregulated in an ox‐LDL‐induced HUVEC model, providing additional biological support for its involvement in CA.

Among the identified proteins, PARP1 plays a central role in DNA repair, inflammation, and oxidative stress [34]. Previous studies have shown that PARP1 activation exacerbates endothelial dysfunction and promotes atherogenesis through increased oxidative damage and inflammatory signaling [35, 36]. Notably, Fan et al. [37] have also highlighted the relevance of inflammatory pathways in cardiovascular disease progression, although they prioritized different targets such as AZGP1 and ALDH2. Our findings extend these observations by suggesting that genetically predicted higher PARP1 levels are associated with a lower risk of CA under the assumptions of MR analyses. Furthermore, a study by Li et al. [38] has elucidated the role of PARP1 in diabetic atherosclerotic calcification. They found that PARP1 deletion attenuates calcification and vessel stiffening in diabetic mice by regulating Stat1‐mediated phenotypic switching of vascular smooth muscle cells and macrophage polarization. In addition, the prioritization of PARP1 was further supported by cross‐platform validation using an independent Icelandic SomaScan pQTL dataset from deCODE genetics, in which PARP1 showed consistent and directionally concordant associations with CA. Although PARP1 was identified as a potentially protective protein in the MR analysis, its upregulation observed in ox‐LDL‐treated endothelial cells may reflect an acute stress‐responsive or compensatory process rather than the long‐term systemic protein exposure represented by genetic proxies [39, 40]. The induction of PARP1 under ox‐LDL stimulation is consistent with its established roles in endothelial stress responses and inflammatory regulation [35, 36]. Given the central contribution of endothelial dysfunction to the initiation and progression of atherosclerosis, these findings provide biological plausibility linking genetically predicted PARP1 regulation to vascular inflammatory processes. Collectively, the convergent evidence from genetic analyses, cross‐platform replication, and preliminary cellular experiments supports the translational relevance of PARP1 as a potential therapeutic target in CA, with implications for future drug repurposing strategies beyond lipid‐lowering therapies [41].

Prior studies have shown that SDCCAG8 is essential for primary cilium integrity and centrosome‐associated processes and that its deficiency disrupts ciliary architecture and cellular signaling [42, 43]. In vascular endothelial cells, cilia‐mediated mechanotransduction plays an important role in shear stress sensing, inflammatory regulation, and vascular homeostasis [44, 45]. Disruption of this process can lead to endothelial dysfunction, a recognized early event in atherogenesis [46, 47]. FST, a known inhibitor of activins within the TGF‐*β* superfamily, showed a potentially protective association with CA in our analysis [48]. Experimental models have shown that FST administration reduces vascular inflammation, intimal hyperplasia, and fibrosis [49]. Furthermore, clinical observational studies have reported lower circulating FST levels in patients with established coronary artery disease [50]. FOLH1, also known as prostate‐specific membrane antigen (PSMA), has been predominantly studied in oncology [51]. However, it is also expressed in nonmalignant tissues and participates in folate metabolism and vascular homeostasis [52]. Previous studies have suggested that impaired folate metabolism is associated with oxidative stress, endothelial dysfunction, and early atherogenesis [53–55]. NCAN is an extracellular matrix proteoglycan involved in tissue remodeling and lipid retention within arterial walls [56]. Previous GWAS meta‐analyses have associated NCAN variants with plasma lipid traits, including triglyceride and LDL‐C levels, but causal links to coronary disease had remained unclear [57]. Moreover, extracellular matrix remodeling, including the role of proteoglycans like NCAN, is increasingly recognized as critical for lipid trapping and plaque stability during atherogenesis [58]. Consistent with previous studies, our genetic findings further suggest the potential involvement of SDCCAG8, FST, FOLH1, and NCAN in CA, supported by MR, colocalization, and SMR/HEIDI analyses, although the strength of colocalization evidence varied across proteins.

From a translational perspective, the identified proteins may provide potential opportunities for both biomarker development and therapeutic intervention. Compared with Fan et al.′s broader cardiovascular disease framework, our CA‐specific focus yielded distinct targets, emphasizing the value of disease‐specific proteomic MR analyses [37]. For example, PARP1 and FST represent inflammation‐ and fibrosis‐modulating pathways particularly relevant to atherosclerotic plaque progression [35, 36, 49]; SDCCAG8 highlights a previously underappreciated mechanosensory and endothelial regulatory pathway [44, 46, 47], whereas FOLH1 and NCAN highlight underexplored metabolic and matrix‐related mechanisms [53, 59]. Moreover, the mechanistic diversity among these proteins suggests the potential for multitarget combination strategies to more effectively address the complex pathophysiology of CA [60].

Importantly, while our study observed strong colocalization signals for PCSK9, a well‐validated clinical target for LDL‐C lowering, it did not meet the stringent SMR and HEIDI criteria for candidate protein prioritization. This finding is consistent with other proteome‐wide MR studies, which have also noted that some clinically validated targets do not always show robust genetic evidence for causality, due to method‐specific assumptions, differences in regulatory architecture, and the complex relationship between circulating protein levels and tissue‐specific functional effects [61, 62]. Notably, PCSK9 regulation involves both hepatic tissue–specific mechanisms and posttranslational processes that may not be fully captured by plasma pQTL‐based SMR/HEIDI frameworks. Consistent with this interpretation, a recent multiomics MR study by Liu et al. [19], incorporating tissue‐specific eQTL data, identified alternative CA‐related targets, rather than PCSK9, further highlighting that different analytical strategies may prioritize distinct yet complementary biological pathways. In contrast to the study by Liu et al. [19], which identified VAMP8, MFGE8, and PDGFD using tissue‐specific eQTL‐based MR at the transcriptomic level, MFGE8 and PDGFD did not reach statistical significance in our plasma pQTL‐based MR analysis. Although VAMP8 showed an initial association in MR, it was not retained after colocalization and SMR/HEIDI analyses due to insufficient evidence supporting a shared genetic signal between protein levels and CA. These discrepancies likely reflect fundamental differences between transcriptomic and proteomic regulation [63]. Collectively, these findings underscore the complementary nature of transcript‐level and protein‐level genetic evidence and highlight the importance of integrating genetic, biological, and clinical evidence when prioritizing therapeutic targets [61, 64].

While this study provides important evidence supporting the prioritization of candidate plasma proteins associated with CA, several limitations should be acknowledged. First, all genetic datasets used in this analysis were derived from individuals of European ancestry, which may limit the external validity of our findings. Given that the prevalence of CA, allele frequencies, LD structures, and genetic determinants of lipid metabolism and inflammatory pathways differ substantially across ethnic groups, the identified protein–CA associations may not be directly generalizable to non‐European populations, such as East Asian or African cohorts [65]. Second, although our findings provide genetic evidence supporting PARP1, SDCCAG8, FST, FOLH1, and NCAN as potential therapeutic targets, cross‐platform validation using the SomaScan pQTL dataset showed that only PARP1 demonstrated a statistically significant and consistent association with CA, highlighting the challenges of cross‐platform reproducibility in proteome‐wide genetic studies. Therefore, the evidence supporting the remaining candidate proteins remains comparatively limited and should be interpreted with caution. Importantly, the absence of SomaScan‐based validation for SDCCAG8 and FST does not necessarily invalidate the Olink‐based findings. Rather, it may reflect intrinsic differences between proteomic platforms, including variations in aptamer‐ and antibody‐based detection technologies, protein coverage, and the availability of eligible cis‐pQTL instruments. In addition, although we performed preliminary functional investigations of PARP1, experimental validation remains essential to elucidate the underlying mechanisms. Third, tissue‐specific expression and posttranslational modifications, not captured in plasma proteomic datasets, could influence protein function in the atherosclerotic microenvironment. Finally, although restricting IVs to cis‐acting pQTLs helped reduce horizontal pleiotropy, this approach may have overlooked relevant trans‐regulatory mechanisms influencing CA. Nevertheless, complementary analyses, including Bayesian colocalization and MR sensitivity tests, supported the robustness of these associations by suggesting that they were unlikely to be driven by LD or directional horizontal pleiotropy. Despite these measures, residual confounding inherent to genetic association studies cannot be entirely excluded. Therefore, systematic functional and pharmacological studies in in vitro and in vivo models of atherosclerosis are warranted to further validate their biological roles, druggability, efficacy, and safety. Integration with multiomics approaches—including transcriptomics, epigenomics, metabolomics, and single‐cell proteomics—will be crucial to map the pathways through which these proteins exert their effects [66, 67]. Longitudinal cohort studies across diverse ancestries are needed to confirm the generalizability of these findings.

## 5. Conclusion

This study systematically prioritized PARP1, SDCCAG8, FST, FOLH1, and NCAN as candidate proteins for CA, supported by MR analysis using UKB‐PPP pQTL data and CA GWAS summary statistics, Bayesian colocalization, and SMR/HEIDI analyses. Among these, PARP1 showed the most consistent evidence across Olink‐ and SomaScan‐based pQTL datasets and was upregulated in an ox‐LDL‐induced HUVEC model, representing a promising candidate for future translational studies. Future work should focus on functional validation, multiomics integration, and mechanistic characterization across diverse populations to further clarify the biological and translational relevance of these proteins in CA.

**Nomenclature**
CA: coronary atherosclerosis
CCK‐8: Cell Counting Kit‐8
ELISA: enzyme‐linked immunosorbent assay
FDR: false discovery rate
FOLH1: folate Hydrolase 1
FST: follistatin
GDF‐15: growth differentiation Factor 15
GWAS: genome‐wide association study
HEIDI: Heterogeneity in Dependent Instruments
HUVECs: human umbilical vein endothelial cells
ICAM1: intercellular adhesion Molecule 1
IL‐1*β*
: interleukin‐1*β*
IL‐6: interleukin‐6
IVs: instrumental variables
LD: linkage disequilibrium
LDL‐C: low‐density lipoprotein cholesterol
MR: Mendelian randomization
NCAN: neurocan
OSM: oncostatin M
ox‐LDL: oxidized low‐density lipoprotein
PARP1: poly(ADP‐ribose) Polymerase 1
PP.H4: posterior probability for H4
pQTLs: protein quantitative trait loci
SDCCAG8: serologically defined colon cancer Antigen 8
SMR: summary‐data‐based Mendelian randomization
TNF‐*α*
: tumor necrosis factor‐alpha
UKB‐PPP: UK Biobank Pharma Proteomics Project
VCAM1: vascular cell adhesion Molecule 1

## Author Contributions

D.G. is responsible for the study design, definition of intellectual content, manuscript preparation, and editing. H.L. is responsible for the data acquisition, analysis, and statistical analysis. S.W. is responsible for the literature research, clinical studies, and experimental studies. Y.W. and H.Y. are responsible for the manuscript review. Z.W. is responsible for the guarantor of integrity of the entire study and study concepts.

## Funding

This study was funded by the Zhejiang Province Health Industry Science and Technology Plan Project (Grant/Award Number: 2025HY0891).

## Disclosure

All authors read and approved the final manuscript.

## Ethics Statement

This study used deidentified, publicly available summary‐level data from the UK Biobank Pharma Proteomics Project, FinnGen consortium, and deCODE genetics dataset. No human participants or animal experiments were involved. No additional ethical approval was required.

## Consent

The authors have nothing to report.

## Conflicts of Interest

The authors declare no conflicts of interest.

## Supporting information


**Supporting Information** Additional supporting information can be found online in the Supporting Information section. Table S1: List of 6202 genome‐wide significant and LD‐independent cis‐pQTL variants used as instrumental variables in the proteome‐wide MR analysis. Table S2: Results of heterogeneity test based on the cis‐pQTL instruments. Table S3: Results of horizontal pleiotropy test based on the cis‐pQTL instruments. Table S4: Leave‐one‐out sensitivity analysis results for MR estimates. Table S5: Single‐SNP sensitivity analyses for protein coronary atherosclerosis associations. Table S6: Posterior probabilities (PP.H0–PP.H4) from Bayesian colocalization analysis for 13 plasma proteins associated with coronary atherosclerosis. Table S7: Summary‐data‐based Mendelian randomization and HEIDI test results for 14 candidate plasma proteins.

## Data Availability

All data generated or analyzed during this study are included in this published article and its supporting information files. Further enquiries can be directed to the corresponding author.
